# Identification and validation of diagnostic biomarkers for intrahepatic cholestasis of pregnancy based on untargeted and targeted metabolomics analyses of urine metabolite profiles

**DOI:** 10.1186/s12884-023-06102-6

**Published:** 2023-11-30

**Authors:** Weici Liu, Lingyan Chen, Keyan Miao, Yilan You, Jingyang Li, Jianfeng Lu, Yan Zhang

**Affiliations:** 1 Wuxi Maternal and Child Health Hospital, Wuxi Medical Center of Nanjing Medical University, Wuxi, Jiangsu 214023 China; 2 The Affiliated Wuxi People’s Hospital of Nanjing Medical University, Wuxi People’s Hospital, Wuxi Medical Center, Nanjing Medical University, Wuxi, Jiangsu 214023 China; 3 Medical College, Soochow University, Suzhou, Jiangsu 215123 China; 4 Wuxi Maternal and Child Health Care Hospital, Wuxi, Jiangsu 214023 China

**Keywords:** Intrahepatic cholestasis of pregnancy, Urine metabolite, Diagnostic biomarker, 3-hydroxypropionic acid, Uracil

## Abstract

**Background:**

Intrahepatic cholestasis of pregnancy (ICP) is a prevalent pregnancy-specific complication that presents with maternal itching and elevated serum bile acid levels. ICP is associated with unfavorable pregnancy outcomes, severely decreasing the pregnant woman’s quality of life. Timely identification of ICP is crucial for effective management and improved outcomes.

**Methods:**

We collected urine samples from 8 patients with ICP and 8 healthy individuals. We used Liquid Chromatography-Mass Spectrometry (LC-MS) to detect metabolite expression levels, then conducted a series of bioinformatic analyses to explore the potential biological meanings of differentially expressed metabolites, and preliminarily discovered several candidate biomarkers. To validate these candidate biomarkers, we performed Gas Chromatography-Mass Spectrometry (GC-MS) detection and analyzed their diagnostic values using receiver operating characteristic (ROC) curve.

**Results:**

Untargeted metabolomics data showed that 6129 positive peaks and 6218 negative peaks were extracted from each specimen. OPLS-DA analysis and the heat map for cluster analysis showed satisfactory capability in discriminating ICP specimens from controls. Subsequent analysis extracted 64 significantly differentially expressed metabolites, which could be potential biomarkers for diagnosis of ICP. Based on the KEGG enrichment analyses, six candidate biomarkers were preliminarily identified. Two most promising biomarkers (3-hydroxypropionic acid and uracil) were validated by targeted metabolomics analyses with the area under the curve (AUC) of 0.920 and 0.850 respectively.

**Conclusion:**

Based on preliminary screening from untargeted metabolomics and subsequent validation through targeted metabolomics, 3-hydroxypropionic acid and uracil were identified as promising diagnostic biomarkers for ICP.

**Supplementary Information:**

The online version contains supplementary material available at 10.1186/s12884-023-06102-6.

## Background

Intrahepatic cholestasis of pregnancy (ICP) is the most common pregnancy-specific liver disease characterized by generalized pruritus that commonly includes the palms and soles, elevated serum total bile acids (TBA), and increased liver transaminases [1–4]. The symptoms occur mostly in the third trimester, but can be earlier in pregnancy [5]. ICP is associated with multiple adverse pregnancy outcomes, including spontaneous and iatrogenic preterm birth, fetal distress, meconium staining of the amniotic fluid, and still birth [1–3]. The risk of complications increases with the level of maternal serum bile acids, so women with more severe cholestasis are at higher risk [6–9]. The incidence rate of ICP generally ranges between 0.2% and 2%, but varies widely with ethnicity and geographic location [1]. The incidence was once reported to be as high as 15.6% among the Araucanos Indians in Chile [10]. On the contrary, a study involving 1 million women in Sweden estimated the incidence to be between 0.32% and 0.58% [8]. In the United States, the reported incidence rate ranges from 0.32% in Connecticut to 5.6% in Los Angeles [11].

The diagnosis of ICP is currently based on pruritus and elevated total serum bile acids, with other hepatobiliary disease excluded [3, 5]. Total serum bile acids concentration has been the most often used biomarkers for the diagnosis of ICP in clinical practice [12]. Most studies use a cutoff value for ICP ranging from 10 to 14 µmol/L, but the diagnostic accuracy is unsatisfactory [1, 3, 12]. The liver transaminases, mainly aspartate aminotransferase (AST) and alanine aminotransferase (ALT), are also commonly increased in ICP, but similarly, their sensitivity and specificity are not satisfying [1, 3]. Alkaline phosphatase (ALP) is usually increased as well, but it is a nonspecific indicator of ICP [1, 3]. Moreover, serum bilirubin is raised in only about 10% of patients [1, 3]. Due to discomforting symptoms and severe complications of ICP, pharmacologic treatment is required to improve pruritus and prevent fetal complications [13]. In recent 20 years, ursodeoxycholic acid (UCDA) is a commonly-used drug that can efficiently alleviate pruritus, improve liver function tests, and prevent poor pregnancy outcomes in women suffering from ICP [3, 4, 13].

As the pathogenesis of ICP is related to abnormal metabolism caused by multiple factors of which genetic susceptibility and reproductive hormones are predominant, serum and urine levels of relevant metabolites alter in ICP patients in contrast with normal people [3, 14, 15]. Thereby, discovery of those differential metabolites is beneficial to establishing diagnostic biomarkers for ICP. Nonetheless, at present, the commonly used diagnostic indicators are several routine inflammatory markers with certain limitations, failing to achieve a high level of accuracy. As a consequence, novel effective biomarkers or combined application of several biomarkers are urgently demanded. Among diverse approaches assessing the metabolites (including mass spectrometry, untargeted metabolomics, targeted metabolomics, and imaging metabolomics), untargeted metabolomics strategy is prioritized for its rapidity, high throughput and sensitivity [16, 17]. Fortunately, several metabolic biomarkers for ICP diagnosis have been reported in previous studies. For instance, Ma et al. discovered a maternal urinary metabolite panel as a non-invasive biomarker for ICP diagnosis and suggested several primary causative factors in ICP pathogenesis [18]. Yurtcu et al. reported that four serum adipokine metabolites had predictive and diagnostic value in identifying ICP patients [19]. Feng et al. uncovered that ICP resulted in remarkable alteration in serum metabolites of neuropeptide Y [20].

In our current research, we analyzed the differentially expressed metabolites in the urine specimens from ICP patients via untargeted metabolomics and preliminarily identified several significantly differentially expressed serum metabolites as promising biomarkers for diagnosis of ICP. Moreover, we conducted targeted metabolomics analyses to validate the diagnostic performances of two metabolites. Finally, we combined the results of both targeted and untargeted metabolomics and identified two underlying diagnostic biomarkers for ICP.

## Materials and methods

This project comprised two successive rounds of experimentation. The initial round centered on untargeted metabolomics research employing Liquid Chromatography-Mass Spectrometry (LC-MS) technology. The experimental procedures encompassed sample metabolite extraction, LC-MS/MS detection, and subsequent data analysis. The data analysis encompassed fundamental analysis and differential expression analysis. The former encompassed data pre-processing, Student’s t-test, principal component analysis (PCA), partial least squares-discriminant analysis (PLS-DA), orthogonal partial least squares-discriminant analysis (OPLS-DA), as well as screening and identification of differentially expressed compounds. The latter encompassed hierarchical clustering analysis, Z-score graphs, correlation heat map analysis, Kyoto Encyclopedia of Genes and Genomes (KEGG) annotation analysis, and metabolomics pathway analysis of the differentially expressed metabolites. The subsequent round relied on Gas Chromatography-Mass Spectrometry (GC-MS) technology for targeted metabolomics research. The experimental procedures primarily entailed sample metabolite extraction, enrichment, and derivation, GC-MS/MS detection, and subsequent data analysis.

### Urine sample collection

All urine specimens were collected from Wuxi Maternal and Child Health Care Hospital (Jiangsu, China) and preserved in the room with a condition of appropriate temperature and humidity. All experimental procedures were approved by the Ethics Committee of Wuxi Maternal and Child Health Care Hospital (protocol code: 2020-01-0310-08). The overview of the patient characteristics is presented in Table 1. Urine specimens collected during delivery from pregnant women were divided into 2 groups, the ICP group (T2) and normal group (N2), each corresponding to 8 biological replicates, for a total of 16 specimens. The ICP specimens were collected from the patients diagnosed with ICP according to the following diagnostic bases——maternal pruritus, abnormal liver function (elevated serum AST and ALT levels), increased TBA concentrations, and exclusionary diagnosis, while the control specimens were acquired from healthy pregnant women. Subsequently, in order to enhance the robustness of our diagnostic validation for potential biomarkers, an additional 4 urine specimens, comprising two ICP and two control specimens, were incorporated into the subsequent targeted metabolomics experiments. In short, 16 and 20 urine specimens were collected for untargeted metabolomics and targeted metabolomics, respectively.

**Table 1:** Demographics and baseline characteristics of the pregnant women

Index	CON (n = 10)	ICP (n = 10)	t/t’ test	P value
Age(year)			0.944	0.356
Mean ± SD.	33.3 ± 5.6	31.4 ± 4.2		
Median(IQR)	33.0(28.0–38.0)	31.5(29.0–34.0)		
Range	25.0–44.0	24.0–39.0		
BMI (kg/m^2^)			0.271	0.789
Mean ± SD.	26.9 ± 3.3	26.6 ± 3.0		
Median (IQR)	27.3(24.9–29.2)	27.2(24.5–28.4)		
Range	20.6–32.9	19.5–31.3		
TBA(µmol/L)			4.940	0.000
Mean ± SD.	3.6 ± 2.2	61.8 ± 40.7		
Median (IQR)	3.0(2.2–4.4)	43.4(35.8–87.1)		
Range	1.4–8.9	20.4–166.0		
AST(µmol/L)			3.501	0.002
Mean ± SD.	18.8 ± 5.6	57.6 ± 38		
Median (IQR)	18.0(14.4–24.0)	55.3(30.5–70.5)		
Range	11.0-27.9	11.1-130.4		
ALT(µmol/L)			3.561	0.002
Mean ± SD.	11.7 ± 7.1	91.8 ± 67.9		
Median (IQR)	9.6(8.3–11.4)	62.3(17.7-144.4)		
Range	6.3–33.3	3.7-212.1		
ALP(µmol/L)			2.140	0.047
Mean ± SD.	167.7 ± 84.2	227.4 ± 47.4		
Median (IQR)	143.7(107.7-189.8)	224.2(183.8-262.9)		
Range	92.7–367.0	158.7-302.3		
Time of onset (day)				
Mean ± SD.		215.6 ± 55.9		
Median (IQR)		237.0(196.0-246.0)		
Range		63.0-266.0		
Time of delivery (day)			6.668	0.000
Mean ± SD.	273.3 ± 2.6	256.8 ± 8.2		
Median (IQR)	273.0(272.0-274.0)	256.5(249.0-266.0)		
Range	269.0-279.0	246.0-267.0		
Gravida(no.)			0.849	0.406
Mean ± SD.	2.6 ± 1.3	3.1 ± 1.6		
Median (IQR)	2.5(1.0–4.0)	3.0(2.0–4.0)		
Range	1.0–5.0	1.0–7.0		
Para(no.)			0.573	0.573
Mean ± SD.	0.8 ± 0.6	0.9 ± 0.8		
Median (IQR)	1.0(0.0–1.0)	1.0(0.0–2.0)		
Range	0–2.0	0–2.0		

Note: 1) The day of onset refers to the day during which TBA elevation was first detected;

2) When the variance of the two groups of data is homogeneous, the t test is used; When two sets of data have uneven variances, the t ‘test is used

3) no., number; SD., standard deviation; IQR, interquartile range

### Procedures for untargeted metabolomics

#### Urine sample preparation

First, 100 µL of each urine specimen was extracted into centrifuge tubes. Then, 300 µL of methanol (*Merck*, Darmstadt Germany) was added to the specimens, mixed thoroughly by vortex oscillation, and ultrasonicated at 4 ℃ for 30 min. The specimens were stored at -20 ℃ for 1 h and then centrifuged at 4 ℃ (15 min, 12,000 rpm). After the centrifugation, take 200 µL of supernatant and add 5 µL of internal standard (1 mg/mL 2-chloro-1-phenylalanine, *GL Biochem*, Shanghai China). Finally, the mixture was transferred to LC vials [21]. And quality control (QC) samples are prepared by mixing equal volumes of extracts from all samples.

#### UHPLC-Q-TOF/MS analysis

Metabolic profiling of urine specimens was analyzed via an analysis platform, LC-MS (Waters, UPLC; Thermo, Q Exactive). The column (Waters, ACQUITY UPLC HSS T3 1.8 *µ*m, 2.1 mm×100 mm) was employed. Urine specimens were separated by chromatography with the column temperature at 40 ℃ and the flow rate of 0.3 mL/min. The mobile phase A consisted of water and 0.05% formic acid (*CNW*, Shanghai China) while the mobile phase B consisted of acetonitrile (*Merck*, Darmstadt Germany). The elution gradient was started initially with 95% A for 1 min, linearly decreased to 5% A at 12 min, maintained for 1.6 min, and then returned to 95% A for 2.4 min of equilibrium. The automatic injector temperature was 4 ℃, and 5 µL of aliquot of each specimen was injected into the column. Time of flight (TOF)/MS was conducted on both positive ion mode and negative ion mode [21].

Electrospray ionization (ESI) source conditions were set as follows: (1) ESI+ : heater temperature, 300 ℃; sheath gas flow rate, 45 arb; aux gas flow rate, 15 arb; sweep gas flow rate, 1 arb; spray voltage, 3.0 KV; capillary temperature, 350 ℃; S-Lens RF level, 30%; (2) ESI- : heater temperature, 300 ℃; sheath gas flow rate, 45 arb; aux gas flow rate, 15 arb; sweep gas flow rate, 1 arb; spray voltage, 3.2 KV; capillary temperature, 350 ℃; S-Lens RF level, 60%. The parameters of scan mode were set as follows: full scan (m/z 70 ~ 1050); data-dependent secondary mass spectrometry (dd-MS2, TopN = 10); resolution, 70,000 (primary mass spectrometry) & 17,500 (secondary mass spectrometry). And collision mode was set as high-energy collisional dissociation (HCD) [21].

#### Original data pre-processing

After acquiring raw UHPLC-Q-TOF/MS data, we made a series of preparation and collation of the raw data for more effective analysis. We only retained peak area data with no more than 50% null value in one group or in all groups. Then we performed missing value recoding on the original data by means of the minimum value one-half method for filling. Finally, to achieve data normalization, we implemented a normalization procedure based on the total ion current (TIC) of each specimen. [21].

#### Metabolomics data analysis

The pre-processed data were converted to the Statistics Analysis files and then processed using SIMCA software (V14.1, Sartorius Stedim Data Analytics AB, Umea, Sweden). Principle component analysis (PCA) was preformed and a heat map of hierarchical clustering analysis was generated as the unsupervised multivariate statistical analysis. Orthogonal partial least squares-discriminant analysis (OPLS-DA) was conducted as a supervised method to identify the significant variables with discriminatory capability, and validity of the OPLS-DA model was corroborated by a permutation test with a 200-time repetition. The variable importance in the projection (VIP) value of each variable from the OPLS-DA model was computed to demonstrate its contribution to the classification. Metabolites with the VIP value > 1 were further analyzed in Student’s t-test at univariate level to evaluate the significance of each metabolite. Metabolites with P value < 0.05 were regarded to have statistical significance [21].

To assess the differentially expressed metabolites, the volcano plot was made for the two groups. Then we performed hierarchical clustering analysis to compare the difference of metabolite expression mode and conducted a Z-score graph to measure the relative contents of metabolites at the same level. What’s more, Pearson correlation coefficient was calculated to analyze the correlation between all metabolites two by two and the results were shown in the correlation heatmap. Furthermore, the Kyoto Encyclopedia of Genes and Genomes (KEGG) annotation analysis as well as the pathway analysis (including enrichment analysis and topological analysis) was conducted for identification of the differentially expressed metabolites.

### Procedures for targeted metabolomics

#### Urine pretreatment

The standards were weighed precisely with an analytical balance and dissolved in water-acetonitrile (V:V = 98:2, containing 0.1% formic acid) to obtain the standard stock solution, the concentration of which was 1 mg/mL. The stock solution was diluted by gradient at concentrations of 100 µg/mL (working solution 1), 10 µg/mL, 1 µg/mL, and 0.1 µg/mL (working solution 2), respectively. Working solution 1 was used to prepare the mixed standard solution and working solution 2 was used to optimize the mass spectrometry conditions. The mixed standard solution concentrations were 50,000 ng/mL, 20,000 ng/mL, 10,000 ng/mL, 5000 ng/mL, 2000 ng/mL, 1000 ng/mL, 500 ng/mL, 200 ng/mL, 100 ng/mL, 50 ng/mL, 20 ng/mL, 10 ng/mL, 5 ng/mL, respectively. The mixed standard solutions were filled into 1.5 mL EP tubes, and 100 µL of the mixed standard and blank solvent of the above gradient concentration were respectively absorbed into GC derived vials and dried in a rapid centrifugal dryer for derivative treatment [22].

#### Metabolite extraction

Samples stored at -80 ℃ were thawed at room temperature. 100 µL of each sample was added to 1.5 mL EP tubes. 300 µL of ice-cold mixture of methanol-acetonitrile (V:V = 2:1, containing 7 isotopes internal standard) was added, and the mixtures were vortexed for 30 s. The whole samples were extracted by ultrasonic for 10 min in ice-water bath and then stored at -20 ℃ for 30 min. The extract was centrifuged at at 4 °C (12,000 rpm) for 10 min and 100 uL of supernatant was transferred into the wide-lined tubes for evaporation drying. QC samples are prepared by mixing equal volumes of extracts from all samples. The 15 mg/ mL of methoxylamine hydrochloride in anhydrous pyridine (80 µL) was placed into the glass derivatization vial containing the residue, vortex agitation was performed for 2 min, and the mixture was incubated in the incubator at 37 °C for 60 min. Subsequently, 80 µL of BSTFA with 1% TMCS and 20 µL n-hexane were added into the mixture, vortex agitation was conducted for 2 min, and then the mixture was incubated at 70 °C for 60 min. The samples were placed at ambient temperature for 30 min before GC-MS/MS analysis [22].

#### GC-MS/MS analysis

The derivative samples were analyzed on a Trace1310 gas chromatography system coupled to a TSQ9000 mass spectrometer with an electron ionization (EI) source (Thermo Fisher Scientific, USA). The specific analysis conditions and methods of gas chromatography experiment are as follows. A DB-5MS fused-silica capillary column (30 m × 0.25 mm × 0.25 μm, Agilent J&W Scientific, Folsom, CA, USA) was utilized to separate the derivatives. The high purity helium (purity not less than 99.999%) was used as the carrier gas at a constant flow rate of 1.2 mL/min through the column. The injector temperature was maintained at 280 °C. Injection volume was 1 µL by splitless mode, and the solvent delay time was set to 4 min. The initial temperature program of the column incubator was set at 50 °C for 0.5 min, and then it was increased at a rate of 15 °C/min to 125 °C for 2 min, 4 °C/min to 210 °C for 3 min, and finally 15 °C/min to 305 °C for 3 min. The specific analysis conditions and methods of mass spectrometry (MS) experiment are as follows. The temperatures of MS quadrupole and EI source were set to 280 and 300 °C, respectively. MS data were acquired using the selective reaction monitoring (SRM) scan mode with a mass scanning range of m/z 40–600. The QC samples were injected and detected at regular intervals (every 10 samples) throughout the analytical run to provide a set of data from which repeatability can be assessed [22].

#### Statistical analysis

After the raw GC-MS/MS data were acquired, the Student’ s t-test was used to perform comparisons between two groups. A P-value < 0.01 was set as the threshold to determine statistical significance. Boxplots showing concentrations of candidate biomarkers in 10 ICP specimens and 10 control specimens were drawn and the receiver operating characteristic (ROC) curves were subsequently designed to display diagnostic performances. R 4.0.4 software (Foundation for Statistical Computing, Vienna, Austria) was used to perform data analyses.

## Results

### Dimension reduction of untargeted metabolomics analysis

**Fig. 1:**
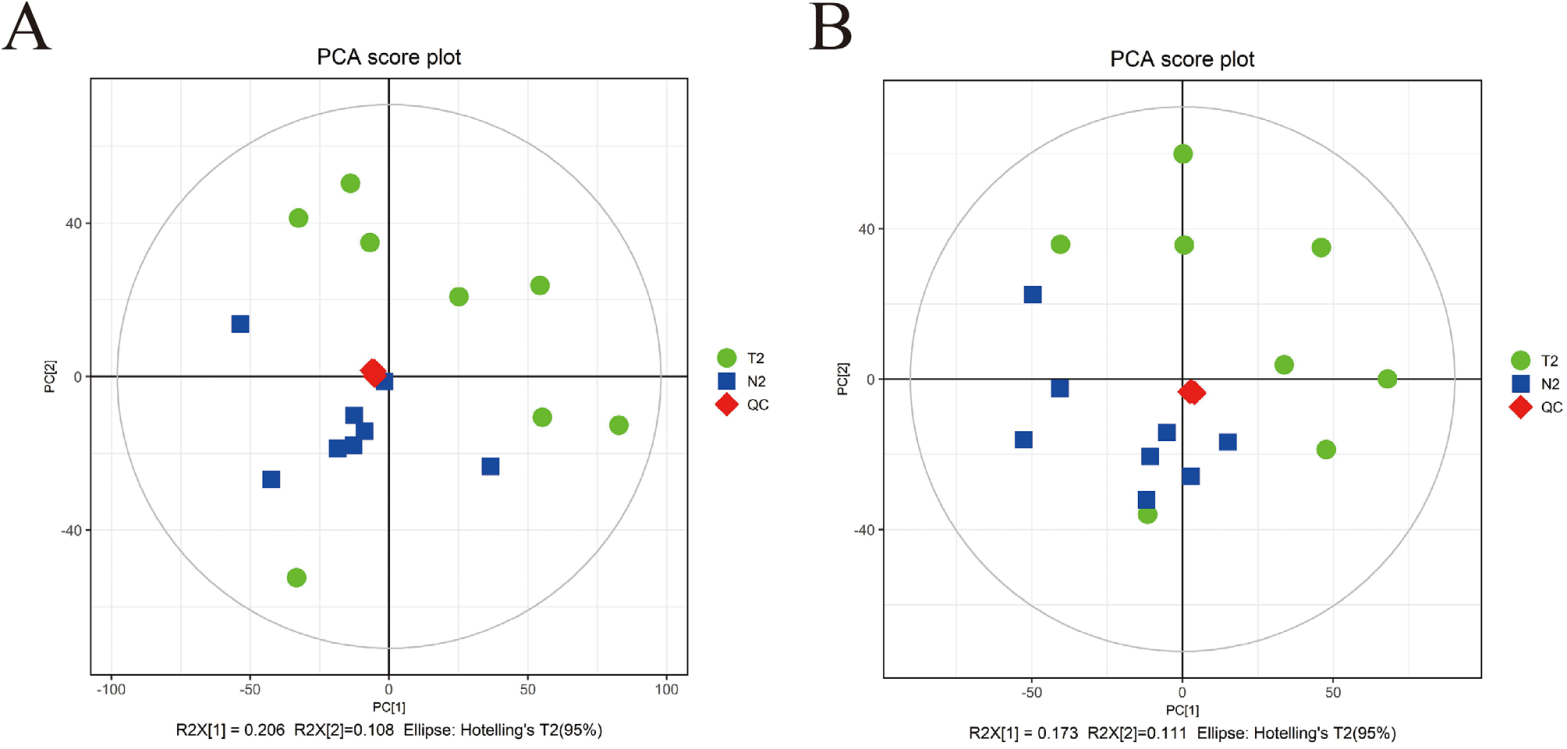
Principle component analysis (PCA) score plots based on the UHPLC-Q-TOF/MS data of urine samples. The distribution of metabolic profiles for the test samples and quality control (QC) samples in PCA. (**A**) Positive ion mode; (**B**) Negative ion mode. N2, normal samples; T2, intrahepatic cholestasis of pregnancy samples

We utilized Pareto scaling and 7-fold cross-validation to realize the establishment of the PCA model according to the molecular characteristics of all the groups from this research, including QC samples. The distribution status of urine metabolites for the test samples as well as QC samples in PCA model was demonstrated in Fig. 1. The QC injections were all clustered intensively in PCA space. The concordance of the iterative QC injections and credible data quality throughout all the specimens unveiled the validity of the metabolic profiling approach during the test . Ultimately, we extracted 6129 positive peaks and 6218 negative peaks in total by means of SIMCA software.

**Fig. 2:**
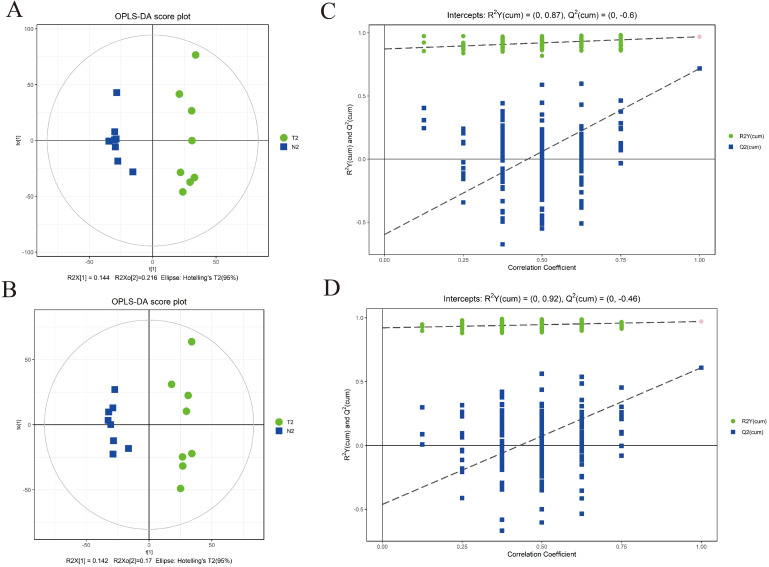
Multivariate statistical analysis of metabolomics between normal and intrahepatic cholestasis of pregnancy (ICP) samples. Orthogonal partial-least squares discrimination analysis (OPLS-DA) score plot derived from UHPLC-Q-TOF/MS-based metabolomics analysis between normal (blue squares) and ICP (green circles) samples. (**A**) Positive ion mode; (**B**) Negative ion mode. Statistical validation of the OPLS-DA model by permutation testing. (**C**) Positive ion mode; (**D**) Negative ion mode. Q^2^, predictive ability; R^2^Y, goodness of fit

We also confirmed the validity of the algorithmic model applied in this research by OPLS-DA. As reflected in the score plots of OPLS-DA model, apparent division between ICP groups and control groups was shown in Fig. 2A and B. Concretely, the explanation rates for Y variable (R²Y) and predictive capability (Q²) were 0.97 and 0.718 in positive ion mode, and 0.971 and 0.609 in negative ion mode, respectively, which corroborated that the OPLS-DA model was stable and reliable. In addition, the cross-validation via permutation and superposition tests (200 times) certified that no models had overfitting performance, supported by specific intercepts of R^2^Y = 0.87, Q^2^ = − 0.6 and R^2^Y = 0.92, Q^2^ = − 0.46 for positive and negative ion mode, respectively (Fig. 2C and D).

### Identification of differently expressed urine metabolites

**Fig. 3:**
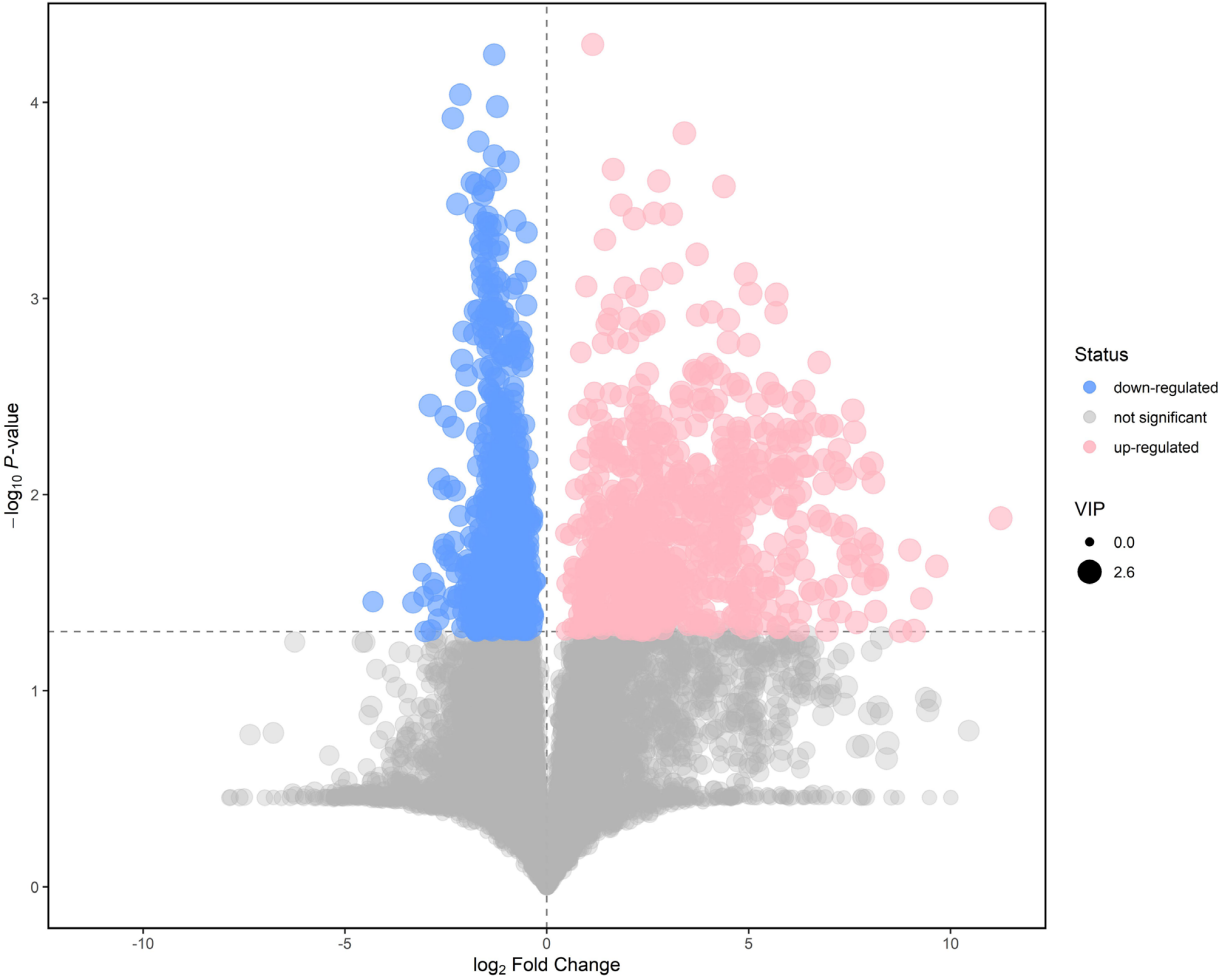
Volcano plot of the differentially expressed metabolites (merged mode). The Y coordinate was -log10 (P value) and the X coordinate was log2 (fold change). Each dot represented a metabolite. Red dots were the upregulated metabolites of significant expression levels. Blue dots were downregulated metabolites of significant expression levels. Black dots were metabolites of non-significant difference. The cutoff standard was set to P value<0.05

Metabolites with the variable importance in the project (VIP) value > 1 were analyzed by Student’s t-test at univariate to examine the differential expression levels. Eventually, 64 differentially expressed metabolites (VIP > 1, P < 0.05) were identified altogether as potential biomarkers for ICP diagnosis. The volcano plot of the differentially expressed metabolites were displayed in Fig. 3. In ICP samples, we discovered that 8 metabolites increased and 28 metabolites declined in positive ion mode (Table 2, Supplemental table). Similarly, 5 metabolites increased and 23 metabolites decreased in negative ion mode (Table 3, Supplemental table). Of all the differentially expressed metabolites, the expression level of sulfochenodeoxycholic acid increased by 242.58 times, exhibiting the most significant alteration in negative ion mode, followed by cholic acid with an increase by 55.97 times.

**Table 2:** Metabolites changed in intrahepatic cholestasis of pregnancy (ICP) urine specimens (positive ion mode)

Name	VIP	P-value	Fold change
**Acetylproline**	2.528	0.001	32.157
** N-Acetylmannosamine**	1.839	0.009	7.482
**3-oxo lithocholic acid**	2.324	0.001	5.429
**19(R)-hydroxy Prostaglandin A2**	1.598	0.036	3.736
**Threonine**	1.778	0.022	3.635
**1-Aminocyclopropanecarboxylate**	1.639	0.047	2.726
**17-alpha-Hydroxyprogesterone**	1.627	0.045	2.415
**Methionine sulfoxide**	1.360	0.037	1.368
**Indole-3-carboxylic acid**	1.434	0.025	0.749
**Adenine**	1.494	0.007	0.714
**2,4-Dihydroxy-butanoic acid**	1.371	0.032	0.708
**cytosine**	1.282	0.038	0.696
**Uracil**	1.513	0.044	0.693
**Indole**	1.386	0.033	0.689
** N-alpha-Acetyl-L-lysine**	1.728	0.015	0.686
**3-Methyladenine**	1.544	0.013	0.657
**Testosterone**	1.517	0.045	0.642
** N,N-Dimethylarginine**	1.804	0.008	0.636
**Creatine**	1.570	0.010	0.634
**Creatinine**	1.732	0.002	0.630
**Glycylproline**	1.684	0.017	0.601
**4-PREGNEN-20-ALPHA-OL-3-ONE**	1.378	0.040	0.587
**3-Hydroxypropionic acid**	1.456	0.034	0.585
**Guanidinoacetate**	1.650	0.020	0.568
**Indolelactic acid**	1.467	0.021	0.557
**Estrone**	1.361	0.048	0.544
**3-Hydroxyphenylacetic acid**	1.628	0.020	0.537
**p-Benzoquinone**	1.532	0.017	0.521
**1-Methylnicotinamide**	1.320	0.030	0.460
**5,6-Dihydrouracil**	2.123	0.002	0.460
**Trigonelline**	1.521	0.034	0.433
**Androstenedione**	1.804	0.004	0.428
**Androsterone**	1.647	0.009	0.405
**p-Coumaric acid**	1.732	0.001	0.310
**Mono-iso-butyl phthalate**	1.472	0.039	0.299
**Cyclo-prolylglycine**	1.741	0.026	0.272

VIP, the variable importance in the projection; Fold change, the ratio of ICP/CON.

**Table 3:** Metabolites changed in intrahepatic cholestasis of pregnancy (ICP) urine specimens (negative ion mode)

Name	VIP	P-value	Fold change
**Sulfochenodeoxycholic acid**	2.484	0.008	242.582
**Cholic acid**	2.254	0.009	55.967
**Glycochenodeoxycholic acid**	1.461	0.043	8.302
**Taurocholic acid**	1.697	0.045	8.039
**Glycocholic acid**	1.849	0.006	3.720
** L-Glutamic acid**	1.340	0.029	0.844
**2-Oxoadipate**	1.400	0.031	0.738
** N-Acetylglutamic acid**	1.399	0.047	0.709
** L-Pyroglutamic acid**	1.465	0.021	0.687
** L-Threonic acid**	1.710	0.004	0.674
**Xanthosine**	1.328	0.047	0.673
**N2-Methylguanosine**	1.536	0.016	0.636
**Mevalonic acid**	1.380	0.019	0.622
**Geranic acid**	1.543	0.043	0.606
** L-Histidine**	1.401	0.041	0.598
**Imidazoleacetic acid**	1.636	0.035	0.559
**DL-4-Hydroxy-3-methoxymandelic acid**	1.447	0.039	0.555
** N-Methylleucine**	1.745	0.028	0.553
**3-Methoxyphenylacetic acid**	1.498	0.047	0.524
**Ethylmalonic acid**	1.634	0.032	0.509
**Perillic acid**	1.762	0.008	0.463
**D-Glutamine**	1.662	0.029	0.450
**2,3-dinor Prostaglandin E1**	2.238	0.000	0.434
**Glycyl-L-leucine**	1.645	0.018	0.432
**Uracil**	1.800	0.002	0.390
** N-acetyltryptophan**	1.252	0.033	0.359
**Hydroxyphenyllactic acid**	1.737	0.023	0.355
**Nonanoic acid**	1.763	0.033	0.284

VIP, the variable importance in the projection; Fold change, the ratio of ICP/CON.

**Fig. 4:**
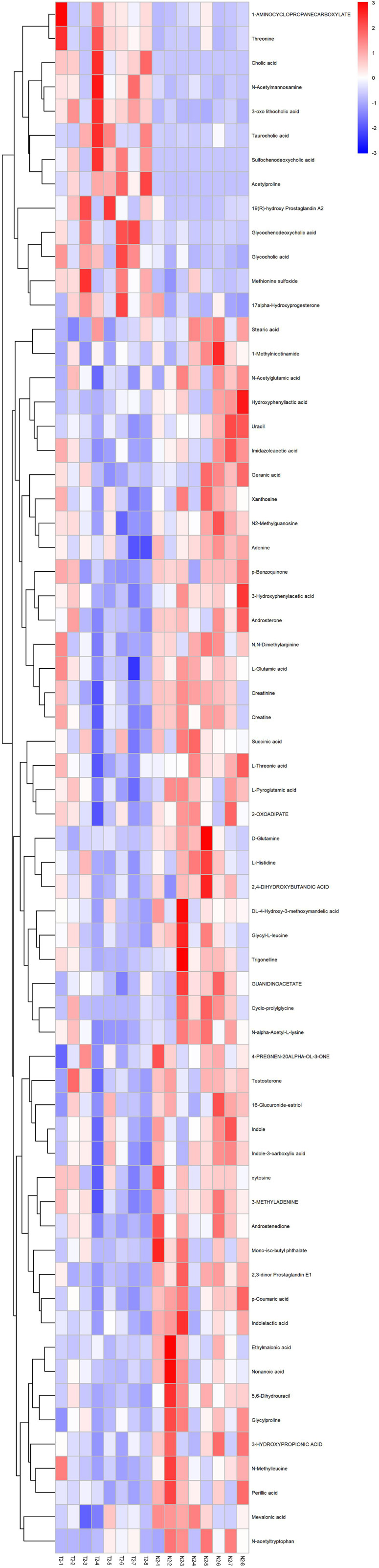
Heatmap of clustering analysis of ICP samples and normal controls (merged mode). Cluster assay was used to analyze the expression of 64 identified metabolites in normal and ICP groups. Blue color was used to indicate decreasing of metabolites in ICP group. Red color was used to indicate increasing of metabolites in ICP group

**Fig. 5:**
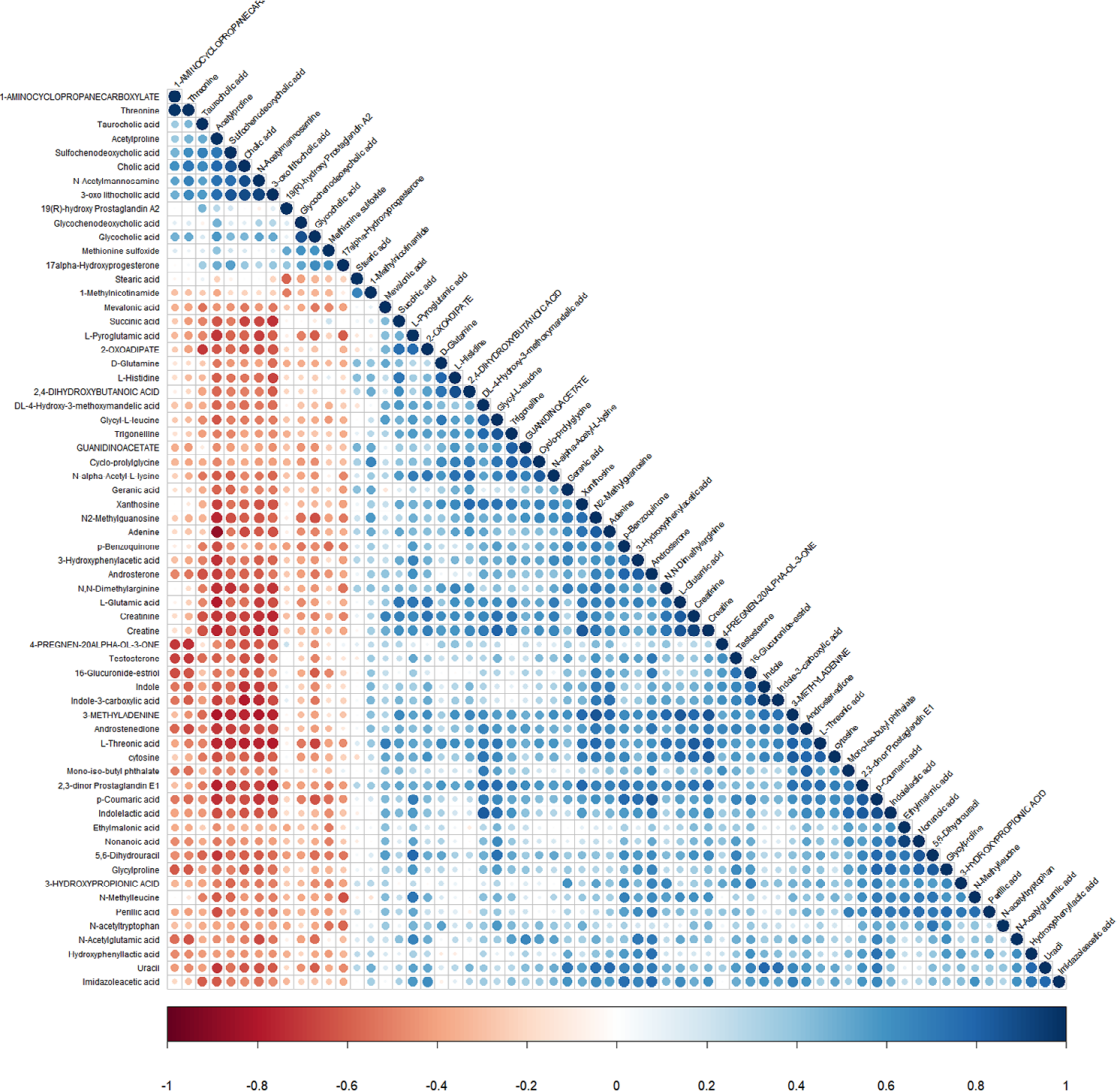
Heatmap of differential metabolite correlation coefficient between ICP group and normal group (merged mode). Pearson correlation coefficients between all metabolites were calculated to analyze the correlation between each metabolite. When the linear relationship between the two metabolites is enhanced, the positive correlation tends to 1 (blue dots), and the negative correlation tends to -1 (red dots). Statistical significance test was conducted for metabolite correlation analysis, and the threshold of significant correlation was selected with significance level P-value < 0.05

Then, we utilized the heat map of hierarchical clustering analysis as another effective strategy to assess the relationship between ICP samples and normal samples and uncover the expression differences of metabolic profiles distinctly. In Fig. 4, we can observe the similarity between the metabolite abundance profiles, displaying a desirable discerning ability between the two groups. Additionally, in order to measure the relative abundance of metabolites at the same level, we computed the Z-scores of the metabolites and draw a graph to display the results (Supplemental Figure S1). To examine the consistency of the trend between two metabolites, we calculated the Pearson correlation coefficient and analyzed the correlation between all metabolites as shown in Fig. 5. In this figure, p-Coumaric acid and androsterone had a strongly positive correlation between their changing trends, indicating a related biological behavior, while adenine and acetylproline had a remarkably negative correlation.

### Pathway analysis of the differentially expressed metabolites

**Fig. 6:**
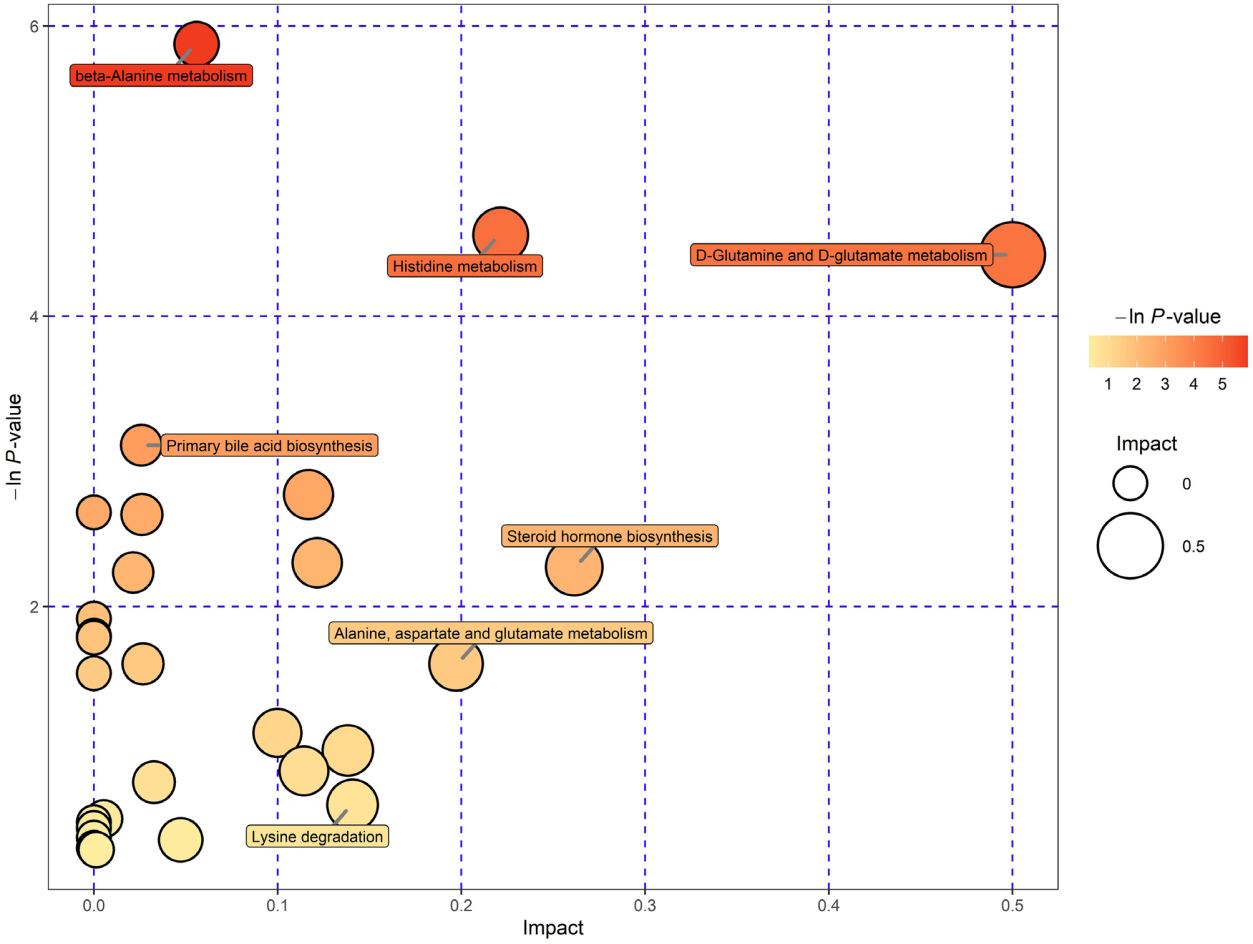
Pathways related to identified metabolites. Kyoto Encyclopedia of Genes and Genome (KEGG) analysis enriched all identified metabolites into 29 different pathways. The pathways with significant P-value were annotated in the figure

With comprehensive analysis (including enrichment analysis and topological analysis), 29 pathways associated with differentially expressed metabolites were observed. Among all pathways, remarkably enriched terms incorporated D-Glutamine and D-glutamate metabolism, histidine metabolism, beta-Alanine metabolism, primary bile acid biosynthesis, steroid hormone biosynthesis, etc. (Fig. 6). Notably, steroid hormone biosynthesis possessed the largest number of overlapped compounds, which could play a crucial role in the pathogenesis of ICP. The differentially expressed metabolites (also named as hit compounds in KEGG pathway analysis) in five remarkably enriched pathways were shown in Table 4, and through the intersection of KEGG analysis results and 64 different metabolites previously screened, six candidate biomarkers were preliminarily identified——3-hydroxypropionic acid, L-histidine, L-glutamic acid, D-glutamine, uracil, and 5,6-dihydrouracil. Taking into account the data in Tables 2 and 3, two differentially expressed metabolites (3-hydroxypropionic acid and uracil) were finally identified as promising diagnostic biomarkers for ICP.

**Table 4:** Hit compounds in five remarkably enriched pathways

Pathway	Total	Hit	P value	Impact	Hit compound
**Beta-Alanine metabolism**	21	4	0.00283	0.0560	3-Hydroxypropionic acid; 5,6-Dihydrouracil; Uracil; L-Histidine
**Histidine** **metabolism**	16	3	0.0105	0.2213	L-Glutamate; L-Histidine; Imidazole-4-acetate
**D-Glutamine and D-glutamate metabolism**	6	2	0.0120	0.5000	L-Glutamate; D-Glutamine
**Primary bile acid biosynthesis**	46	4	0.0446	0.0259	Cholic acid; Glycochenodeoxycholate; Glycocholate; Taurocholate
**Steroid hormone biosynthesis**	85	5	0.1030	0.2615	17-alpha-Hydroxyprogesterone; Testosterone; Androstenedione; Androsterone; 16-Glucuronide-estriol

### Validation of diagnostic performances of two candidate biomarkers

**Fig. 7:**
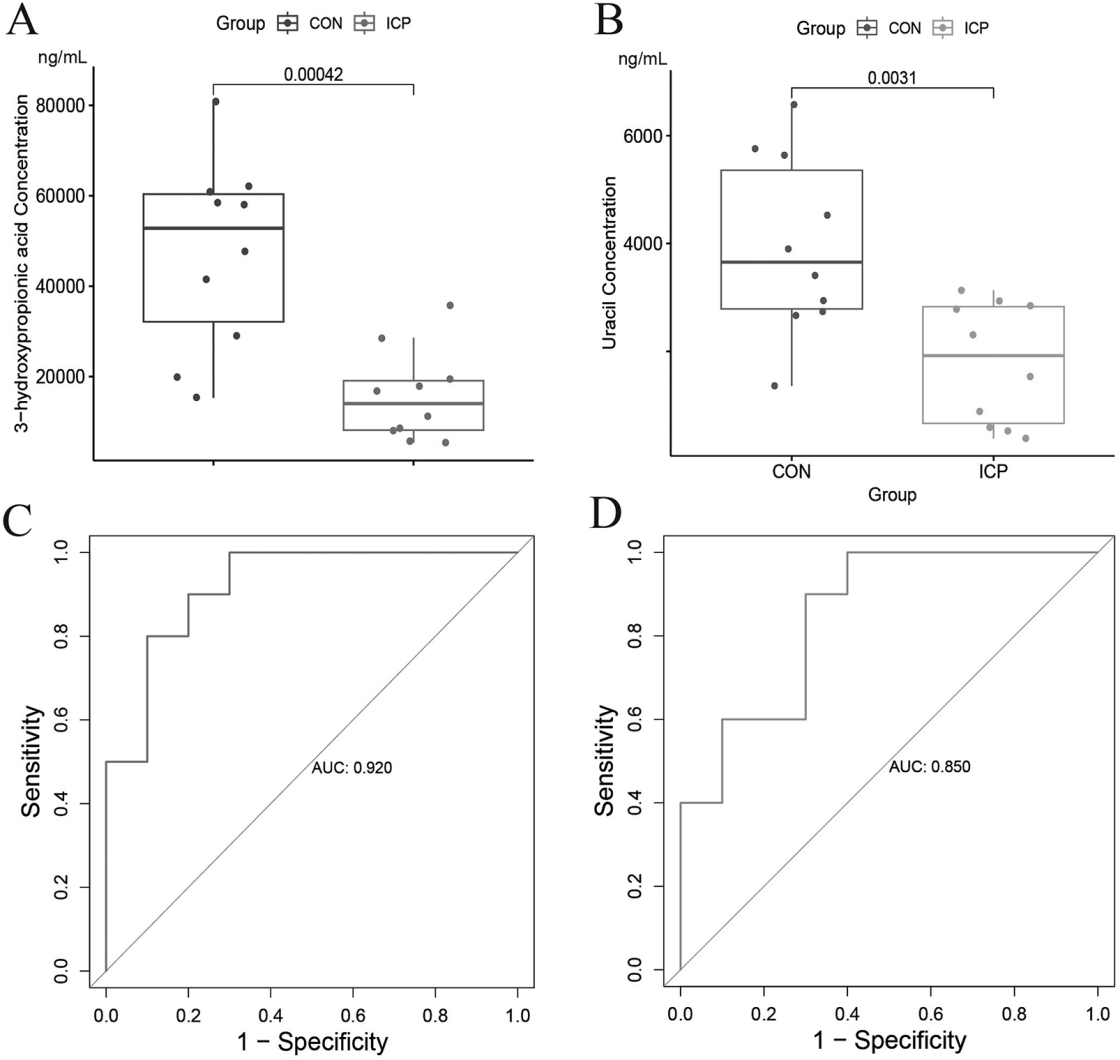
Validation of diagnostic performances of two candidate biomarkers. Boxplots showing concentrations of (**A**) 3-hydroxypropionic acid and (**B**) uracil in 10 ICP specimens and 10 control specimens. The ROC curve analysis for the diagnostic performances of (**C**) 3-hydroxypropionic acid and (**D**) uracil. ICP, intrahepatic cholestasis of pregnancy; CON, control group; ROC curve, receiver operating characteristic curve

The quality control analysis result showed the high degree of curve overlap in total ion current diagram, indicating the consistency of retention time and peak intensity of technical duplicate samples collected in different time periods, as well as good signal stability of mass spectrometry and liquid phase system (Supplemental Figure S2). Based on the GC-MS/MS analysis, we obtained the concentrations of 3-hydroxypropionic acid and uracil in all urine specimens, discovering that the average concentration of 3-hydroxypropionic acid in the ICP group was significantly lower than that in the control group with fold change of 0.332 and P-value < 0.01 (Fig. 7A; Table 5). Comparable findings were presented in uracil with fold change of 0.454 and P-value < 0.01 (Fig. 7B; Table 5). The receiver operating characteristic (ROC) curve analysis was subsequently conducted to display the diagnostic performances of 3-hydroxypropionic acid and uracil. In Fig. 7C and D, the area under the curve (AUC) of 3-hydroxypropionic acid and uracil was 0.920 and 0.850 respectively, showing relatively ideal diagnostic performances. The specific cutoff value, sensitivity, specificity, and Youden index were given in Table 6. Actually, we conducted the GC-MS/MS experiment to analyze a metabolite panel consisting of 100 acid metabolites, with 3-hydroxypropionic acid and uracil included. Several remarkably downregulated acid metabolites were screened as candidate biomarkers with fold change < 0.5 and P-value < 0.05 (Table 5).

**Table 5:** Potential diagnostic biomarkers for ICP in targeted metabolomics

Metabolite	Ave (ICP)/(ng/mL)	Ave (CON)/(ng/mL)	Fold change	P-value
**3-hydroxypropionic acid**	15732.548	47372.174	0.332	0.0004
**Uracil**	1793.620	3950.872	0.454	0.0031
**Glycolic acid**	29776.145	61405.950	0.485	0.0144
** L-malic acid**	1034.511	2442.886	0.423	0.0340
**DL-pyroglutamic acid**	37181.635	72246.583	0.515	0.0310
** L-4-hydroxyproline**	1360.906	2802.899	0.486	0.0362
** L-phenylalanine**	1128.042	2467.921	0.457	0.0018
**cis-aconitate**	49167.892	98772.967	0.498	0.0293
**Citric acid**	66582.475	141568.053	0.470	0.0069
**Homogentisic acid**	46.536	120.449	0.386	0.0245
**3-(4-Hydroxyphenyl)lactate**	298.919	582.549	0.513	0.0125
**Palmitic acid**	467.207	1113.219	0.420	0.0223
**Linoleic acid**	74.395	195.675	0.380	0.0043

Ave (ICP), the average concentrations of metabolites in ten urine specimens from ICP pregnant women; Ave (CON), the average concentrations of metabolites in ten urine specimens from healthy pregnant women; Fold change, the ratio of Ave (ICP)/Ave (CON); ICP, intrahepatic cholestasis of pregnancy; CON, the control group

**Table 6:** Diagnostic criteria for 3-hydroxypropionic acid and uracil for ICP

Biomarker	Cutoff value (ng/mL)	Diagnostic sensitivity	Diagnostic specificity	Youden index	AUC
**3-hydroxypropionic acid**	19681.936	0.800	0.900	0.7	0.920
	28799.566	0.900	0.800	0.7	
**uracil**	2938.360	0.900	0.700	0.6	0.850
	3271.682	1.000	0.600	0.6	

## Discussion

With the progression in high-throughput sequencing technologies (e.g. gene chips) and high-performance liquid chromatography, it is easier for investigators to study diagnostic biomarkers [23, 24]. In the meantime, technological breakthroughs accelerate the concept of multi-omics to come into researchers’ minds, and multi-faceted research of the transcriptome, proteome, and metabolome may motivate us to more profoundly explore the functions and mechanisms of these biomarkers [25]. Good news is that an increasing number of novel biomarkers are preliminarily discovered to have a relatively idea performance in diagnosing ICP, supported by trials of clinical diagnostic accuracy. Three lncRNAs (ASO3480, ENST00000505175.1, and ENST00000449605) were identified by Zou et al. from 143 differentially expressed lncRNAs, remarkably downregulated in the ICP group [26]. The combined use of these three lncRNAs is a more credible tool to diagnose ICP and may be a complement for TBA diagnosis [26]. Dong et al. reported that peroxisomal acyl-CoA oxidase 1 (ACOX1), L-palmitoylcarnitine, glycocholic acid, and their combination were promising biomarkers for the diagnosis and prediction of ICP during the first-, second- and third-trimester by means of metabolomics and proteomics [4]. In a pseudo-targeted metabolomics study conducted by Cui et al., a promising combination biomarker consisting of α-MCA, TCA, and Gtri-8 was uncovered to possess an idea diagnostic performance (AUC = 0.996, YI = 0.940) and to be superior to serum TBA for ICP diagnosis [27].

Metabolomics is the scientific study of the complete set of small molecules, or metabolites, present in an organism’s cells, tissues, or biofluids. These metabolites encompass a wide range of compounds including lipids, amino acids, sugars, nucleotides, and others [16]. Metabolomics aims to comprehensively analyze and understand the dynamic changes in these metabolites in response to various biological processes, environmental factors, and diseases [28]. There are two main approaches in metabolomics: targeted metabolomics and non-targeted metabolomics. Targeted metabolomics involves the quantification of a predefined set of specific metabolites or metabolic pathways. Researchers select a subset of metabolites relevant to their research question and use high-sensitivity analytical techniques, such as mass spectrometry, to precisely measure the concentrations of these selected metabolites. This approach is often employed when the focus is on known pathways or biomarkers, allowing for accurate and specific quantification. It provides precise quantification of selected metabolites and is suitable for detecting low concentrations of metabolites [29]. Untargeted metabolomics aims to comprehensively profile the entire metabolome without prior selection of specific metabolites [30, 31]. This approach utilizes high-resolution analytical techniques, such as mass spectrometry coupled with chromatography, to detect and identify a wide range of metabolites, including both known and unknown compounds. So it enables the detection of known and unknown metabolites, providing a holistic view. At the same time, it generates large amounts of data requiring complex analysis and may not offer the same level of accurate quantification as targeted approaches. Targeted and Untargeted metabolomics approaches can complement each other. Targeted metabolomics can offer precise quantitative information about specific metabolites, while untargeted metabolomics provides a broader overview and the potential to discover novel metabolites. Additionally, targeted metabolomics can be used to validate findings from untargeted metabolomics, confirming changes in specific metabolites or pathways.

In our untargeted metabolomics analysis, the most significant pathways are beta-alanine metabolism, histidine metabolism and D-Glutamine and D-glutamate metabolism according to the P-value of the KEGG pathway analysis. Being the only naturally occurring non-essential beta amino acid, beta-Alanine is metabolized by carnosine and acts as a buffer in the cell [32]. Beta-Alanine metabolism starts with a product of aspartate metabolism and then undergoes phosphorylation through an ATP-driven pantothenate kinase [33]. Pantothenate, vitamin B5, is a precursor for synthesis of 4’-phosphopantetheine moiety of coenzyme A and acyl carrier protein [34, 35]. Histidine biosynthesis is inherently linked to the pathways of nucleotide formation. Histamine arises in many tissues by the decarboxylation of histidine, which in excess causes constriction or dilation of various blood vessels, which may be related to postpartum hemorrhage in some ICP patients. Histidine is also a precursor for carnosine biosynthesis (via carnosine synthase), with beta-alanine being the rate limiting precursor [36]. It is a very important amino acid in keeping a moderate pH in the body, as it acts as a shuttle for protons to maintain a balance of acids and bases in the blood and different tissues [36, 37]. D-glutamic acid is naturally found primarily in the cell wall of certain bacteria [37]. Glutamine is the most abundant free amino acid in circulating and intracellular library [38]. It works in many biology syntheses: substrate for protein synthesis, anabolic precursor for muscle growth, substrate for hepatic and renal gluconeogenesis, precursor for glutathione production, etc. [39]. Glutamine plays a central role in fetal carbon and nitrogen metabolism and act as a substrate to meet the growing demand for ATP, biosynthetic precursors and reducing agents in dividing cells [38, 40]. Specific amino acid transporters allow glutamine to enter the cell, which is then converted into glutamate in the mitochondria, which is a precursor to the TCA cycle intermediate alpha ketoglutaric acid [41]. The increase of glutamate metabolites may indicate the increase of energy expenditure requirement during the development of ICP.

In our targeted metabolomics analysis, 3-hydroxypropionic acid and uracil were validated as promising biomarkers, implying that some urine metabolites which might reflect the metabolic characteristics are gradually being recognized. 3-hydroxypropionic acid (3-HP), a three-carbon organic acid with a hydroxyl group on the second carbon, is an intermediate metabolite during propionate metabolism in the human body. 3-HP has been investigated for its potential as a biomarker for metabolic disorders, such as nocturia [42] and its diagnostic and prognostic values in disease management deserve further exploration. However, no study has reported a clear relationship between 3-HP and ICP. Uracil belongs to the family of nucleotide bases, which are the building blocks of DNA and RNA. In humans, uracil is primarily found in RNA, where it pairs with adenine to form the base pairs that make up the RNA molecule. Uracil can be used as a biomarker in many diseases, including cancers, infections, and metabolic disorders. Specifically, the presence of uracil in urine or plasma can indicate DNA damage and abnormal cell growth and some researchers have proposed that uracil can be utilized in prostate cancer diagnosis [43]. Similarly, the detection of uracil in urine or blood can help to diagnose and monitor the progression of infection such as the clinical diagnosis of multiple traumas complicated with sepsis [44]. Also, the level of uracil in urine or blood can function as an indicator to diagnose and monitor the treatment of hereditary orotic aciduria [45]. Although our research revealed 3-HP and uracil had relatively ideal diagnostic performances, this still requires further validation from larger sample-size trials. The combined examination of novel biomarkers and traditional inflammatory biomarkers has been demonstrated to enhance diagnostic performance [46], so the level of 3-HP (or uracil) should be combined with levels of TBA, AST, ALT, or ALP for further validation in future work. What’s more, our targeted metabolomics experiment suggested several extra underlying biomarkers (Table 5) that demand further verification due to their insignificant changes in the untargeted metabolomics experiment.

This study has three limitations. First, our experiment was designed as a controlled study with a small patient number of only 10 ICP specimens and 10 control specimens. On condition that more specimens are acquired, our experiment will end up with more satisfactory results. Second, we only analyzed the urine specimens. The metabolites in the urine can be affected more or less by the renal processing. If serum specimens can be obtained, the results of our study may be more precise and meaningful. Last, our research is lacking in profound analysis of ICP pathogenesis from a metabolic perspective. Mechanistic studies are still in infancy, deserving deeper exploration in future work.

## Conclusion

Through preliminary screening from untargeted metabolomics and validation from targeted metabolomics, 3-hydroxypropionic acid and uracil were identified as promising diagnostic biomarkers for ICP with AUC of 0.920 and 0.850 respectively. Nonetheless, this experiment was performed as a controlled study with a small sample size. The diagnostic performances of 3-hydroxypropionic acid and uracil still demand more convincing validation from multicenter clinical trials with large sample sizes in future research.

### Electronic supplementary material

Below is the link to the electronic supplementary material.

Supplementary Material 1

Supplementary Material 2

## Data Availability

The underlying data can be obtained from the corresponding author (Yan Zhang).
