# Supplementary material for: Identification and validation of diagnostic biomarkers for intrahepatic cholestasis of pregnancy based on untargeted and targeted metabolomics analyses of urine metabolite profiles
Source: BMC Pregnancy Childbirth. 2023 Nov 30;23:828. doi: 10.1186/s12884-023-06102-6 (PMC10691115; doi:10.1186/s12884-023-06102-6)
Supplement: Supplementary file 1 — Supplementary Material 1 [file 12884_2023_6102_MOESM1_ESM.docx]

**Supplemental figure titles and legends**


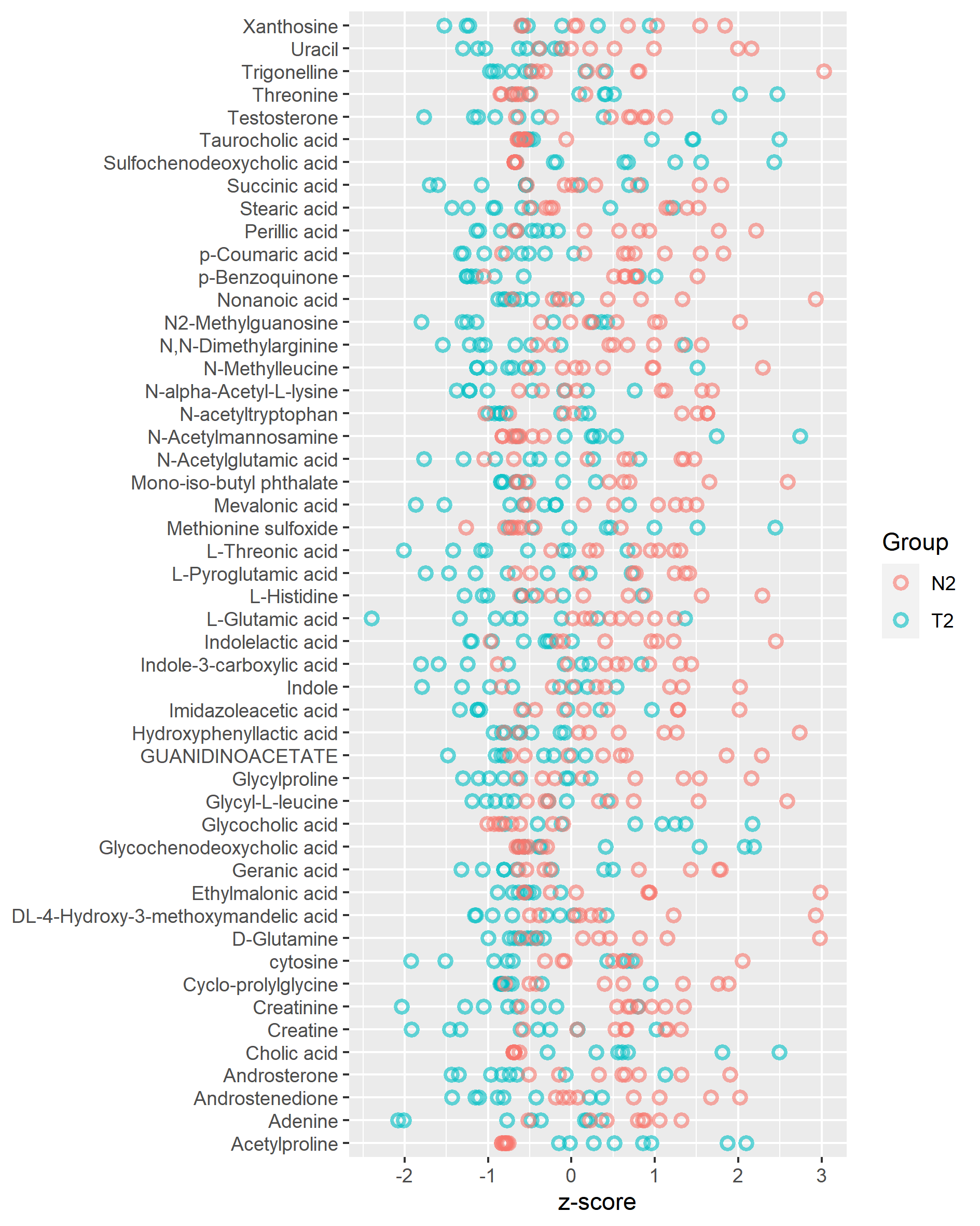


Supplemental Figure S1. Z-scores of the metabolite profile in urine specimens.


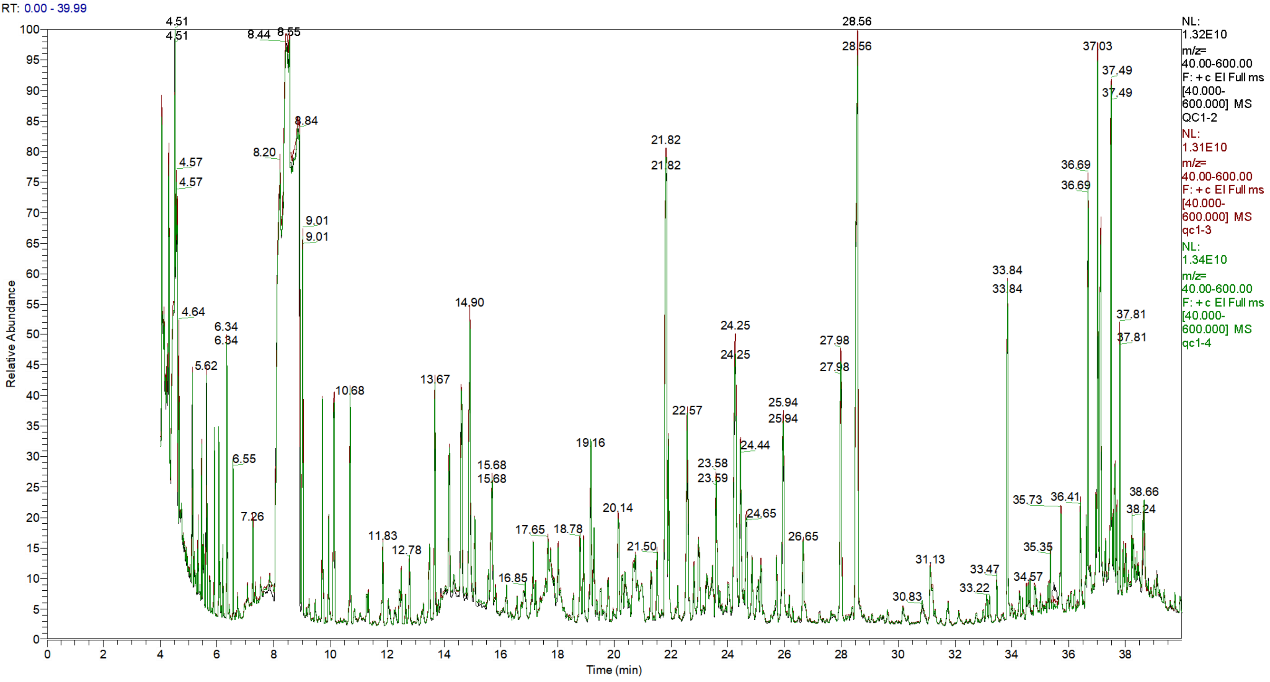


Supplemental Figure S2. Total ion current diagram (quality control sample superposition).
